# Commentary to Nicastro et al. (2016), Borderline Personality Disorder and Emotion Dysregulation

**DOI:** 10.1186/s40479-016-0045-1

**Published:** 2016-11-22

**Authors:** Nader Perroud, Ueli Kramer

**Affiliations:** 1 University Hospitals of Geneva and University of Geneva, Geneva, Switzerland; 2 Department Psychiatry-CHUV, University of Lausanne, Place Chauderon 18, CH-1003 Lausanne, Switzerland; 3 Department of Psychology, University of Windsor, Windsor, Canada; 4 McLean Hospital, Department of Psychiatry, Harvard University, Belmont, USA

We realized that the paper by Nicastro et al. [3] did not discuss all the studies involving the French version of the BSL-23. Parallel to Nicastro and colleagues’ translation, Kramer et al. [2] have used a slightly different French translation of the BSL-23 which had previously received approval by the authors of the scale (M. Bohus, personal communication, July 2010). This independent translation differs only on 4 items - a matter of nuance - from the Nicastro and colleagues translation. In their treatment study, Kramer and colleagues randomized *N* = 85 patients with Borderline Personality Disorder (BPD) and administered the BSL-23 pre and post treatment for *n* = 61 patients. They found, for a psychiatric treatment over 10 sessions, a small, but significant, pre-post effect (*d* = .28, intent to treat). In a different randomized controlled trial for *N* = 31 patients with BPD, the same author group found a small, but significant, between-group effect favoring a short-term version of dialectical-behavior skills training (*d* = .23; completers; [1]). Given the interest of the BSL-23 in French speaking samples of individuals with BPD, the current validation study by Nicastro et al. [3] is highly welcome and will help encourage the use of the scale in further psychotherapy studies.
